# Orbital apex syndrome from middle meningeal artery embolization

**DOI:** 10.3389/fneur.2026.1915486

**Published:** 2026-08-21

**Authors:** Andrew Trippiedi, Andrew F. Fischer, Michael Dattilo

**Affiliations:** Department of Ophthalmology, Emory University School of Medicine, Atlanta, GA, United States

**Keywords:** case report, diplopia, middle meningeal artery (MMA), Middle meningeal artery embolization (MMAE), orbital apex syndrome

## Abstract

**Purpose:**

To present two cases of orbital apex syndrome, a previously unreported complication of middle meningeal artery (MMA) embolization.

**Background:**

MMA embolization is increasingly used to treat chronic subdural hematomas and is generally considered safe. However, anastomotic connections to the ophthalmic circulation may permit complications from embolic migration.

**Methods:**

Retrospective case series of two patients who developed neuro-ophthalmic complications following MMA embolization for subdural hematoma.

**Results:**

A 75-year-old male developed acute diplopia, a relative afferent pupillary defect (RAPD), and ophthalmoplegia shortly after MMA embolization. Magnetic resonance imaging demonstrated embolic material in the posterior orbit. He was diagnosed with an orbital apex syndrome and treated with intravenous steroids with improvement in ocular motility and alignment and resolution of his RAPD. A 44-year-old male presented 1 year after MMA embolization with progressive diplopia, ptosis, and visual loss. Examination revealed an optic neuropathy, and multiple cranial neuropathies consistent with an orbital apex syndrome. He was treated with oral steroids and had improvement in his vision and oculomotor examination.

**Conclusions:**

Case reports of ophthalmic complications from MMA embolization have reported transient cranial neuropathies or isolated ischemic events, presumably through unintended migration of embolic material through under-recognized meningo-ophthalmic anastomoses. These cases are the first report of orbital apex syndrome as a potential complication of MMA embolization.

## Introduction/Background

The orbital apex represents a critical anatomical junction between the orbit and the intracranial cavity. It is composed of the optic canal and the superior orbital fissure and is in close proximity to the inferior orbital fissure and cavernous sinus. The neurovascular structures essential for visual and ocular motor function, including the optic nerve (cranial nerve (CN) II), ophthalmic artery, oculomotor nerve (CN III), trochlear nerve (CN IV), abducens nerve (CN VI), and the ophthalmic division of the trigeminal nerve (CN V1), along with sympathetic fibers, pass through these confined osseous channels. The dense concentration of structures within this narrow space renders the region particularly vulnerable to compressive, inflammatory, infectious, vascular, and neoplastic processes.

Orbital apex syndrome is characterized by dysfunction of the optic nerve in combination with other cranial nerves (CN III, IV, VI, and V1). Clinically, patients may present with decreased visual acuity, a relative afferent pupillary defect (RAPD), ophthalmoplegia, ptosis, diplopia, or sensory loss in the V1 distribution. Due to the anatomical continuity between the orbital apex, the superior orbital fissure, and the cavernous sinus, orbital apex syndrome can resemble both a superior orbital fissure syndrome and cavernous sinus syndrome. However, optic nerve involvement distinguishes orbital apex syndrome from superior orbital fissure syndrome, and the absence of involvement of the maxillary division of the trigeminal nerve (CN V2) and sympathetic pathway findings help differentiate it from cavernous sinus pathology. (1) Given its strategic location at the interface of the orbit and skull base, pathology at the orbital apex requires prompt recognition and careful radiographic evaluation, as delayed diagnosis may result in permanent visual and ocular motility deficits.

Middle meningeal artery (MMA) embolization is a minimally invasive endovascular procedure that has become an increasingly important treatment for chronic subdural hematomas. Previously, chronic subdural hematomas were managed with observation for mild cases, or surgical evacuation with burr hole drainage for symptomatic or larger collections. Recurrence rates after surgery remained substantial and were thought to be due to persistent bleeding from fragile, inflammatory neovascular membranes within the subdural space. These membranes derive a large portion of their vascular supply from branches of the MMA, which led to the development of MMA embolization as a treatment strategy (2).

MMA embolization is performed via a trans-arterial approach, often through radial or femoral access. Liquid embolic agents or particles are used to occlude distal branches of the MMA supplying the subdural neovascular membranes. Clinically, MMA embolization is used as both an adjunct to surgical evacuation and as a stand-alone therapy in patients with higher surgical risk. Recent randomized studies have shown that adjunctive MMA embolization significantly reduces treatment failure compared to surgical stand-alone therapy, with reportedly no increase in short-term complications (3).

MMA embolization carries a recognized risk of visual complications due to anastomotic connections between the MMA and the ophthalmic arterial systems. Ophthalmic artery occlusions, as well as CN VI palsies causing diplopia secondary to inadvertent embolization of the lacrimal artery, have been reported as complications (4, 5). More recently, cadaveric studies have demonstrated that orbital branches of the MMA, specifically the meningo-lacrimal anastomosis which links the MMA to the infraorbital vasculature through the lacrimal artery, may be more prevalent than initially thought (6). These anatomical connections can provide a path for embolic material to enter the orbit from the MMA.

## Description

We present two male patients, age 75 and 44, who underwent MMA embolization and subsequently developed orbital apex syndromes.

**Figure 1:**
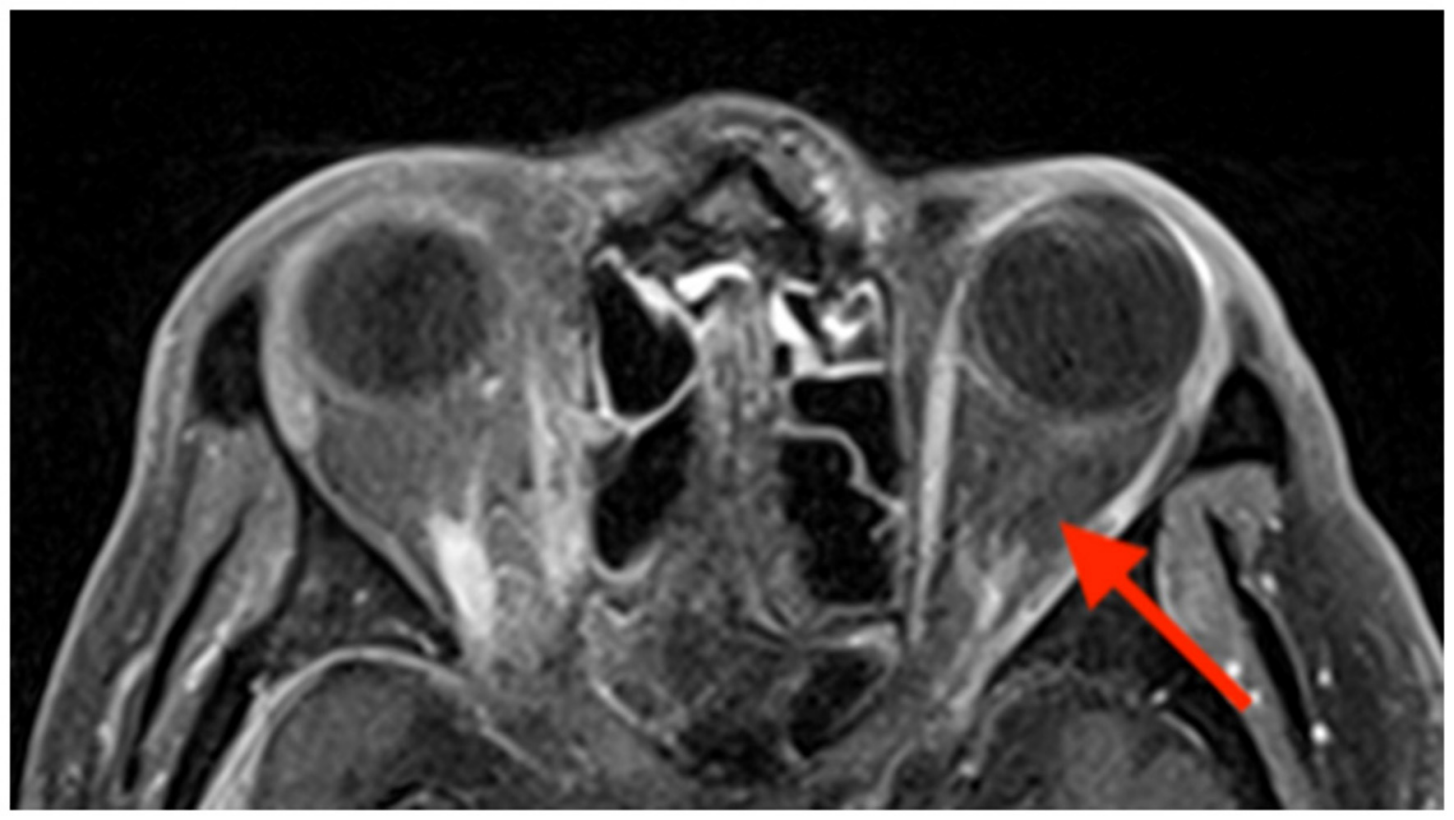
Axial T1 post contrast image with fat suppression from case 1. Arrow points to embolization material.

Case 1: A 75-year-old male presented with upper extremity weakness. CTA head and neck revealed a right sided subdural hematoma measuring up to 2.6 cm with an associated midline shift. He subsequently underwent burr hole evacuation and consecutive MMA embolization. During embolization, embolization material was noted to travel distally from the right frontal branch into both the right parietal and left frontal middle meningeal artery branches. As the embolic material approached the left orbit, embolization was immediately terminated to minimize the risk of off-target embolization. After the MMA embolization, he experienced vertical diplopia and left eyelid swelling. Neuro-ophthalmology examination showed a visual acuity of 20/30 OD and 20/30 OS, a left relative afferent pupillary defect (RAPD), a comitant 6 PD right hypertropia, and supraduction and adduction limitation OS, consistent with a left optic neuropathy and partial third nerve palsy concerning for a left orbital apex syndrome. MRI brain and orbits with and without contrast revealed high density foci suggestive of radio-opaque foreign bodies located along the left lacrimal gland in the posterior and superior orbit, likely representing embolization material (Figure 1). He was initiated on IV methylprednisolone for 3 days with improvement in his extraocular movements. At his 2-month follow-up, examination showed a visual acuity of 20/40 OD and 20/30 OS, no RAPD, full extraocular motility bilaterally, and no ocular misalignment.

**Figure 2:**
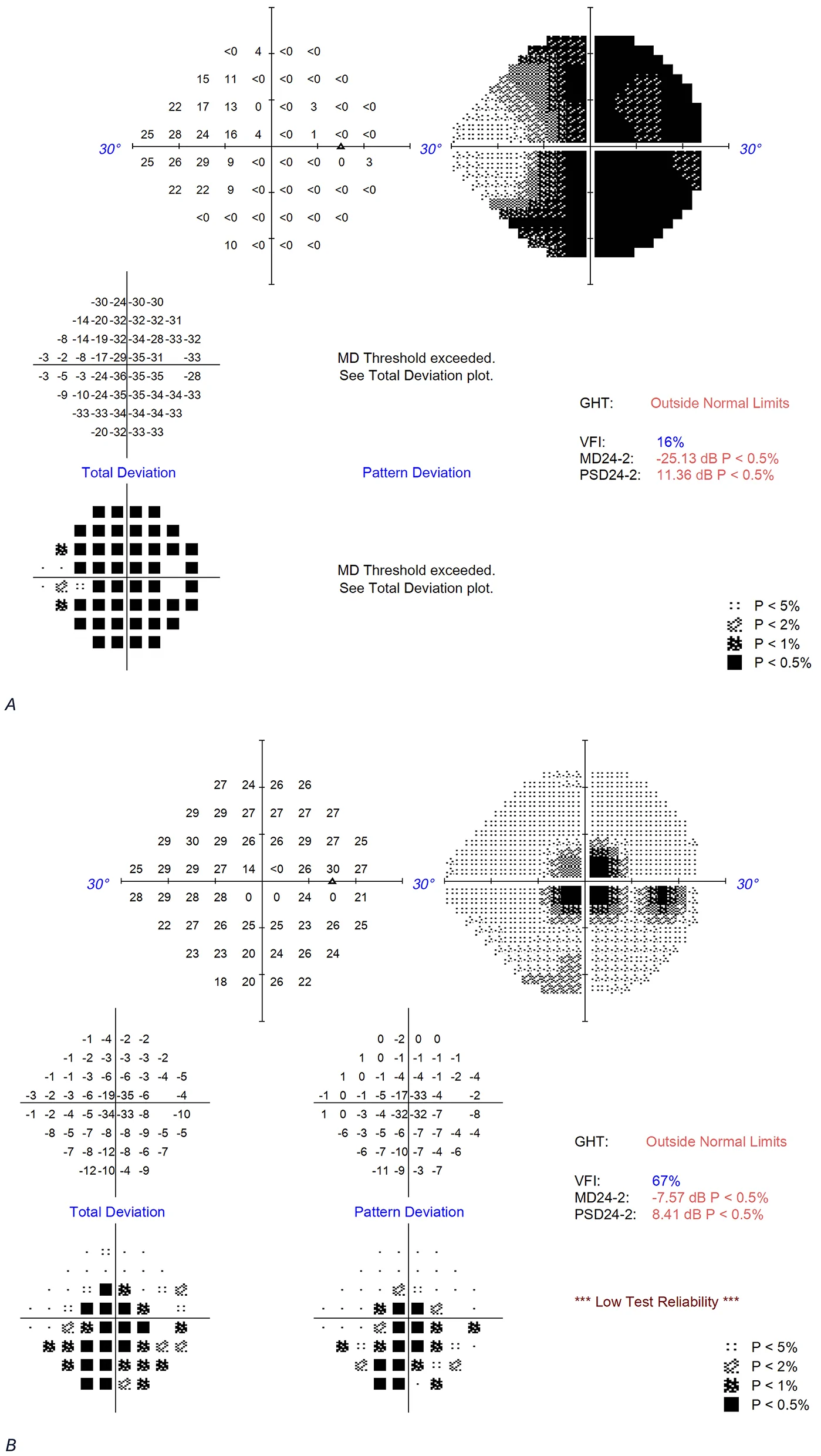
Humphrey visual field (HVF) of the right eye of the patient from case 2. **(A):** HVF on presentation. **(B):** HVF at most recent follow up appointment (10 months after visual field from 2A).

**Figure 3:**
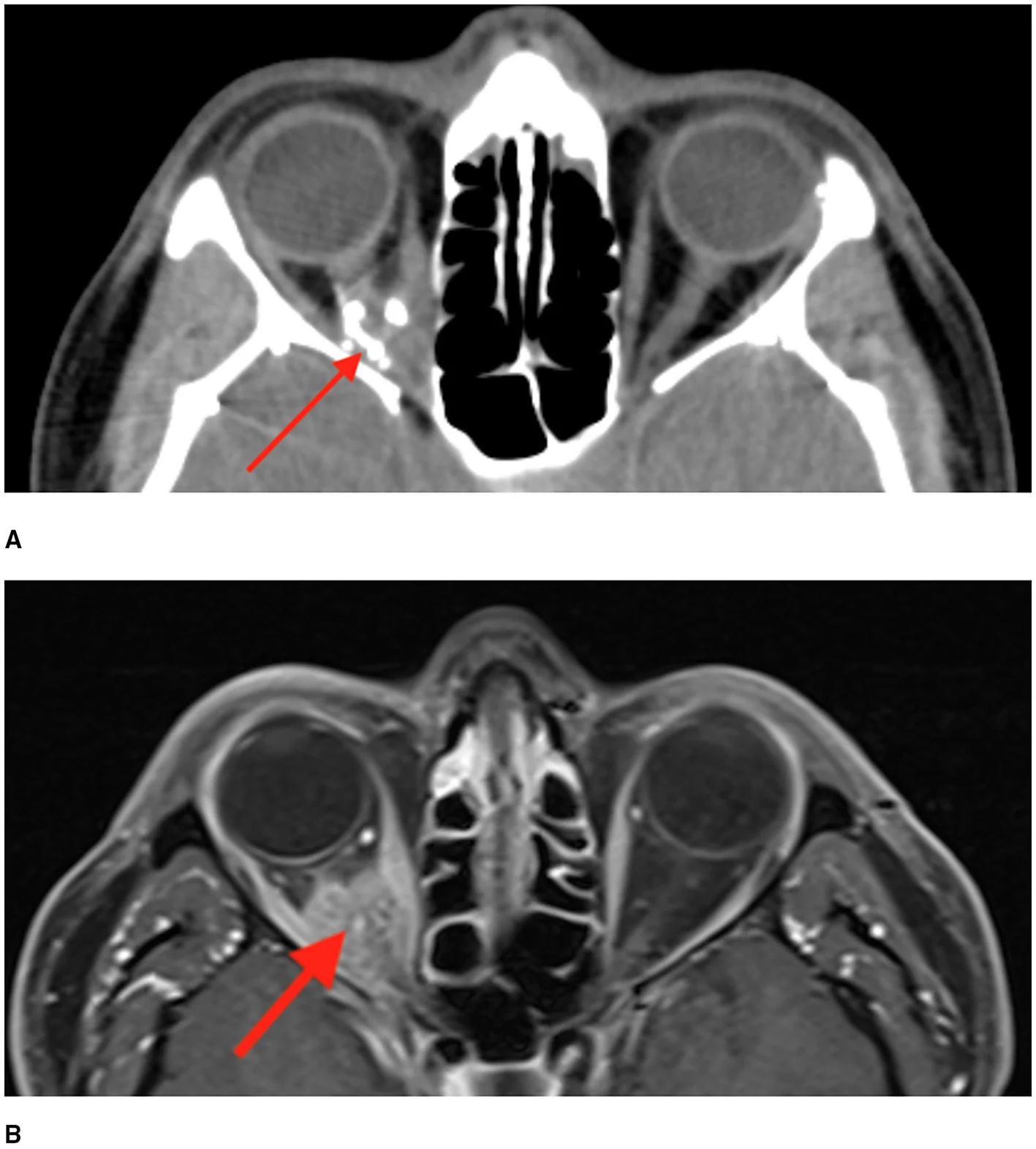
**(A)** CT Orbits without contrast from case 2 obtained 1 year after embolization. Arrow points to suspected embolization material. **(B)** MRI orbits axial T1 post contrast image with fat suppression obtained approximately 1 year after CT scan from 3A. Arrow Points to enhancement compatible with granulomatous reaction to embolization material.

Case 2: A 44-year-old male presented with an acute subdural hemorrhage requiring surgical evacuation and MMA embolization. Approximately 1 year later, he experienced progressively worsening diplopia and right upper eyelid ptosis. CT head without contrast was obtained which showed extension of embolization material into the right orbit with progressive increased soft tissue attenuation within the right orbital apex. His vision ultimately declined, and he was referred to neuro-ophthalmology, where examination showed a visual acuity of 20/125 OD and 20/20 OS, a right RAPD, limited ductions in all directions OD, full ductions OS, and pallor of the right optic nerve. Although he had a left hypertropia in up-gaze, a right hypertropia in downgaze, and a right exotropia in left gaze, he was orthophoric in primary gaze. Humphrey visual field testing revealed a small nasal island of vision OD (Figure 2). His examination was consistent with a right orbital apex syndrome; his optic nerve pallor was presumed to be from his chronic granulomatous process. CT Orbits without contrast revealed embolization material in the right orbit and repeat MRA and MRI brain and orbits with and without contrast showed soft tissue enhancement at the right orbital apex around the optic nerve which was thought to be compatible with a granulomatous reaction to embolization material (Figure 3). He was treated with a 4-week oral steroid taper; his vision improved to 20/60 OD and his strabismus improved and was continued off steroids. At his 2-month follow-up, his visual acuity was 20/150 OD, and he was restarted on 20 mg of prednisone per day. 3 months later, his visual acuity OD had improved to 20/30 (HVF in Figure 2 is shown following this visit). Despite his improvement, he had significant side effects from prednisone including mood lability, and his prednisone dose was decreased to 10 mg daily. He was referred to rheumatology for consideration of long-term immunosuppression.

## Discussion

Orbital and ocular complications from MMA embolization remain rare but have been increasingly recognized as MMA embolization is being performed more commonly. The most frequently reported complication has been transient diplopia with those patients having a nearly complete spontaneous recovery over time (7). More severe ophthalmic complications have also been reported, including central retinal or ophthalmic artery occlusions from migration of embolization material into the ophthalmic circulation (4).

The underlying pathology of these complications is likely from migration of embolization material through arterial anastomotic connections. Indeed, anatomic studies have shown that anastomotic connections between the meningeal and menigo-lacrimal arteries are more common than previously thought and may allow inadvertent migration of embolization material into the orbit despite careful angiographic technique (8, 9). In our first case, review of the procedural angiographic report demonstrated passage of embolic material across the midline into the contralateral frontal MMA branch with embolic material approaching the left orbit, prompting immediate termination of embolization. Although no angiographically visible anastomosis was identified, these findings support inadvertent migration of embolic material distal collateral pathways which may not be fully appreciated angiographically. His 1-line worsening of visual acuity on follow up is likely due to a worsened cataract that was noted on follow up examination which with resolution of his RAPD may account for this decrease in vision.

Previously reported cases of transient diplopia have primarily involved a CN VI palsy, likely related to these anastomotic channels. While the lateral rectus muscle receives its primary blood supply from the lacrimal artery, the remaining extraocular muscles are supplied predominantly by the superior and inferior muscular branches of the ophthalmic artery; the inferior rectus and inferior oblique muscles also receive collateral supply from the infraorbital artery (5, 8–10). In addition, there have been anatomic studies which have also suggested that patients with chronic subdural hematomas have a higher prevalence of clinically significant meningo-ophthalmic anastomoses. The presence of these anatomic variants may represent a relative contraindication to MMA embolization (8, 9).

Although transient cranial neuropathies or isolated ischemic vision loss from MMA embolization have been previously reported, cases of orbital apex syndrome have not been previously reported; both of our patients developed an optic neuropathy and multiple cranial neuropathies consistent with an orbital apex syndrome. Our cases also demonstrate other unique findings, including persistent neurological deficits, improvement with steroid treatment suggesting an inflammatory process, and, in one case, a delayed and progressive course with an associated granulomatous inflammatory reaction. These findings expand the currently reported ocular and orbital complications of MMA embolization to include compressive or inflammatory processes.

## Conclusion

In summary, we describe two cases of orbital apex syndrome following MMA embolization. While previous literature has documented isolated central retinal artery occlusion or a transient cranial nerve VI palsy after MMA embolization, to our knowledge, orbital apex syndrome as a complication of MMA embolization has not been previously described. Therefore, these cases highlight another potential ocular complication of MMA embolization.

## Data Availability

The original contributions presented in the study are included in the article/supplementary material, further inquiries can be directed to the corresponding author.
